# Continuous wound infusion of local anesthetic and steroid after major abdominal surgery: study protocol for a randomized controlled trial

**DOI:** 10.1186/s13063-015-0874-z

**Published:** 2015-08-14

**Authors:** Dario Bugada, Manuela De Gregori, Christian Compagnone, Carolina Muscoli, Ferdinando Raimondi, Silvia Bettinelli, Maria Antonietta Avanzini, Lorenzo Cobianchi, Andrea Peloso, Marco Baciarello, Concetta Dagostino, Luigino A. Giancotti, Sara Ilari, Filomena Lauro, Stefania Grimaldi, Ennio Tasciotti, Massimo Fini, Gloria M R Saccani Jotti, Tiziana Meschi, Guido Fanelli, Massimo Allegri

**Affiliations:** Department of Surgical Sciences, University of Parma, Via Gramsci, 14, 43100 Parma, Italy; SIMPAR Group (Study in Multidisciplinary Pain Research), Parma, Italy; Pain Therapy Service, IRCCS Foundation Policlinico S. Matteo, Pavia, P.le Golgi 19, 27100 Pavia, Italy; Department of Anesthesia, ICU and Pain Therapy, University Hospital of Parma, Via Gramsci, 14, 43100 Parma, Italy; Department of Health Science, Interregional Research Center for Food Safety & Health (IRC-FSH), University “Magna Graecia” of Catanzaro, Complesso “Ninì Barbieri”, 88021 Roccelletta di Borgia, Italy; IRCCS San Raffaele Pisana, Via di Val Cannuta, 247, 00166 Rome, Italy; Department of Anesthesia, IRCCS Humanitas Research Center, Via Alessandro Manzoni, 56, 20089 Rozzano, Milano Italy; Department of Anesthesia and ICU, IRCCS Foundation Policlinico S. Matteo, Pavia, P.le Golgi 19, 27100 Pavia, Italy; Laboratory of Transplant Immunology/Cell Factory, IRCCS Foundation Policlinico “San Matteo”, P.le Golgi 19, 27100 Pavia, Italy; Department of Surgical Sciences, IRCCS Foundation Policlinico S. Matteo, Pavia, P.le Golgi 19, 27100 Pavia, Italy; Department of Nanomedicine, The Methodist Hospital Research Institute, 6670 Bertner Avenue Suite R10-116, Houston, TX 77030 USA; Department of Biomedical, Biotechnological & Translational Sciences (S.Bi.Bi.T), University of Parma, Faculty of Medicine, Via Volturno, 39, 43121 Parma, Italy; Department of Clinical and Experimental Medicine, University of Parma, Via A. Gramsci, 14, 43100 Parma, Italy

## Abstract

**Background:**

Inflammatory response is one of the key components of pain perception. Continuous infusion (CWI) of local anesthetics has been shown to be effective in controlling pain and reducing postoperative morphine consumption, but the effect of adding a potent anti-inflammatory drug (such as a steroid) has never been addressed. In our study, we want to investigate the effect of CWI with local anesthetic + methylprednisolone on acute and persistent pain, correlating clinical data with biomarkers of inflammation and genetic background.

**Methods/Design:**

After approval by their institutional review board, three hospitals will enroll 120 patients undergoing major abdominal surgery in a randomized, double-blind, phase III study. After a 24-h CWI of ropivacaine 0.2 % + methylprednisolone 1 mg/kg, patients will be randomly assigned to receive either ropivacaine + steroid or placebo for the next 24 h. Then, patient-controlled CWI with only ropivacaine 0.2 % or placebo (according to the group of randomization) is planned after 48 h up to 7 days (bolus 10 ml, lock-out 1 h, maximum dose of 40 ml in 4 h). Morphine equivalent consumption up to 7 days will be analyzed, together with any catheter- or drug-related side effect. Persistent post-surgical pain (PPSP) incidence will also be investigated. Our primary endpoint is analgesic consumption in the first 7 days after surgery; we will evaluate, as secondary endpoints, any catheter- or drug-related side effect, genotype/phenotype correlations between some polymorphisms and postoperative outcome in terms of morphine consumption, development of the inflammatory response, and incidence of PPSP. Finally, we will collect, in a subgroup of patients, wound exudate samples by micro-dialysis, blood samples, and urine samples up to 72 h to investigate local and systemic inflammation and oxidative stress.

**Discussion:**

This is a phase III trial to evaluate the safety and efficacy of wound infusion with steroid and local anesthetic. The study is aimed also to evaluate how long this infusion has to be maintained in order to maximize effectiveness. Our data are intended to quantify the amount of ropivacaine and methylprednisolone needed by patients undergoing major abdominal surgery, to be stored in a new nanotechnology device for sustained pain treatment after surgery. We also aim to clarify the roles of inflammatory response, oxidative stress, and genetic background on postoperative and persistent pain after major abdominal surgery.

**Trial registration:**

The trial was registered on ClinicalTrials.gov (NCT02002663) on 24 Oct. 2013.

## Background

Acute postoperative pain is a complex unresolved phenomenon sustained by various patho-physiological changes, potentially leading to postoperative morbidity and persistent post-surgical pain (PPSP) [1], influencing patient outcome [2–4]. Risk factors for PPSP have not been completely established yet, but the severity of acute postoperative pain is often mentioned [5]. Therefore, postoperative pain relief is gaining further interest in the perspective of an improved long-term outcome after hospital discharge.

Postoperative pain treatment involves different strategies; the paradigm of multimodal analgesia is to combine different techniques and drugs to improve efficacy and reduce side effects. The most challenging target is the reduction of opioid consumption because, beyond the well-known respiratory, cognitive, and digestive side effects, opioids may be associated with immune depression and distant malignant cell proliferation and expansion after oncologic surgery [6, 7].

In this perspective, continuous wound infusion (CWI) has gained increasing interest in the last decade. CWI prolongs the action of local anesthetics, improving their efficacy, and has been shown to be effective in controlling pain and in reducing postoperative morphine consumption in major surgery [8]. A further advantage of CWI is the ability to provide a continuous block of pain stimuli coming from the periphery, thus continuously providing protection from central sensitization and pain persistence: the incisional pain model has clearly demonstrated that single-administration, pre-incision analgesia (i.e., preemptive analgesia in clinical practice) is of little interest because when the effects of analgesic treatment abate, the wound itself is able to restart sensitization processes. Therefore, the question is how long continuous infusion should be maintained after surgery.

Nevertheless, postoperative pain is due to different components, and the inflammatory reaction and its non-resolution are now considered to be important factors underlying both acute pain and pain persistence after surgery [1, 9, 10]. A reasonable approach to reduce the inflammatory response is to modulate its local expression with anti-inflammatory agents. Despite the studies that have investigated the efficacy of wound infusion with non-steroidal anti-inflammatory drugs and despite proofs of clinical analgesic efficacy [11], it is well known that cyclooxygenase (COX)-induced enzymes account only partially for local inflammation and that several other substances—especially cytokines and interleukins (ILs)—are involved in the process [12] together with oxidative stress [13]. Although the relative importance of each cytokine may differ depending on the type of surgery, the pro-inflammatory IL-1-β seems to play a major role in incisional pain: it is required for the induction and maintenance of incisional pain in animal models and may contribute to the variation of postoperative morphine consumption in humans [14]. Thereby, the modulation of local cytokine production might represent an attractive alternative for the future developments of intrawound analgesia [15], and the administration of a steroid could be more promising than non-steroidal anti-inflammatory drug infusion [16].

Despite the importance of analgesia, the genetic contribution is considered a determinant for PPSP development, and different studies have highlighted the role of genetic factors on postoperative outcome in terms of analgesic response and pain intensity [17, 18].

Literature data demonstrate that the gene codifying for the adrenergic receptor α-2 (*ADRB2*), a target for adrenaline, is characterized by some single-nucleotide polymorphisms (SNPs) predisposing patients to chronic pain [19]. Diatchenko et al., genotyping 202 females, found that particular *ADRB2* haplotypes modulate chronic pain conditions such as temporomandibular disorder [20].

Also, μ opioid receptor-1 (*OPRM1*) encodes the main receptor of opioids, and its polymorphisms are correlated to a significant interindividual difference in the postoperative consumption of morphine [21–23]. The catecholamine-O-methyltransferase (*COMT*) gene (encoding the enzyme that regulates the levels of catecholamines and enkephalins) has been associated with the variability of pain sensitivity in experimental, chronic, and postoperative pain [17, 20, 24]. The UDP-glucuronosyl-transferase 2B7 gene (*UGT2B7*) is of particular importance for the metabolism of morphine [25], but there are still very limited studies of the association between polymorphic gene expression, opioid treatment doses, and side effects.

Genetics could also be a predictor of the inflammatory response. The interleukin-1 receptor antagonist (IL-1Ra) is the main determinant of the bioactivity of IL-1, and the corresponding gene is polymorphic: variants of this gene have recently been demonstrated to be associated with differences in opioid consumption in the first 24 h after nephrectomy [26].

In our phase III study, we will investigate the analgesic effect of CWI with local anesthetic and methylprednisolone on acute pain and PPSP in the context of open abdominal surgery. Clinical data will be correlated with biomarkers of inflammation and to the genetic background in order to investigate (1) the role of both inflammatory mediators and oxidative stress in the genesis and maintenance of pain and (2) the contribution of gene polymorphisms on inter-individual differences in pain, response to analgesics, and inflammatory reaction after surgery.

## Methods

### Design

This is a multicenter, prospective, double-blind, phase III clinical trial. Three centers are involved in enrollment (IRCCS Policlinico S. Matteo, Pavia, Italy; Azienda Ospedaliero-Universitaria di Parma, Parma, Italy; and Humanitas Research Center, Milano, Italy), and two centers will be in charge of genetic analyses (Azienda Ospedaliero-Universitaria di Parma) and oxidative stress analyses (IRCCS San Raffaele Pisana, Rome, Italy).

The present study is part of a wider project approved by and funded with a grant by the Italian Health Ministry (“New nanotechnology and biomedical approaches to improve postoperative pain treatment reducing risks related to opioids” - GR-2010-2318370; Principal Investigator Massimo Allegri).

This clinical study is locally approved by the institutional review boards of promoting and participating centers (Comitato di Bioetica – Fondazione IRCCS Policlinico S. Matteo; Comitato Etico per Parma – Azienda Ospedaliero-Universitaria di Parma; and Comitato Etico indipendente – IRCCS Istituto Clinico Humanitas; reference number PT-SM-13-YR). The trial is registered on ClinicalTrials.gov (NCT02002663; Principal Investigator Massimo Allegri).

### Patients

Patient eligibility is assessed at the pre-operative anesthesia consultation. All eligible patients must sign a specifically designed informed consent form.

Male and females, American Society of Anesthesiologists I-II-III, from 18 to 85 years old, scheduled to use patient-controlled analgesia (PCA) with morphine for postoperative pain control after major abdominal surgery by laparotomy (gastric surgery, bilious-pancreatic surgery, hepatic resections, bowel resections, nephrectomy, or any laparotomy with intra-peritoneal access) are enrolled.

Regular use of opioid analgesics, history of drugs or alcohol abuse (or both), postoperative hospitalization in intensive care with sedation or mechanical ventilation (or both), neurological disorders, any heart conduction disease, any cognitive or mental disorder hindering a patient from providing informed consent, body mass index of more than 30, diabetes (type I or II), allergy to study drugs, and use of epidural analgesia are exclusion criteria.

### Procedures - clinical study

On the day of surgery, patients come to the operating room and are provided with standard monitoring (electrocardiogram, oxygen saturation, non-invasive blood pressure, and invasive pressure monitoring). General anesthesia is given using propofol and midazolam (as deemed appropriate by the anesthesiologist), opioids (fentanyl 0.2 μg/kg or remifentanil 0.1-0.25 mg/kg per min or both), and muscle relaxants (cisatracurium/rocuronium) and maintained with sevoflurane.

A morphine bolus of 0.15 mg/kg is given 30 to 45 min before the end of surgery. An infusion catheter (Baxter Painfusor®; Baxter International, Deerfield, IL, USA) is placed by the surgeon in the fascial plane between peritoneum and fascia trasversalis, and a 10-ml bolus of 0.2 % ropivacaine is administered immediately after muscular plane closure; the catheter is then connected to an electronic pump (CADD®-Solis; Smiths Medical, Dublin, OH, USA) to give a continuous infusion of pain medications.

During the first 24 h, all patients receive ropivacaine 0.2 % + methylprednisolone 1 mg/kg, 10 ml/h (total volume of 240 ml in 24 h) continuous wound infusion; additionally, either acetaminophen 1000 mg or ketorolac 30 mg every 8 h is prescribed.

### After 24 h, patients are randomly assigned between two groups of treatment:

- Treatment group: 10 ml/h continuous infusion of ropivacaine 0.2 % + methylprednisolone 1 mg/kg
- Placebo group: 10 ml/h continuous infusion of saline 0.9 %.

Regardless of treatment group, rescue analgesia in the first 48 h is provided by PCA pump with morphine (0.5 mg/ml, bolus 1 mg, lock-out 5 min, 20 mg limit every 4 h).

Random group allocation of patients is made through a computer-generated sequence. To maintain blinding, a research fellow who is not taking part in patient evaluation is in charge of preparing and programming all of the pumps used during the study: an opaque, sealed, sequentially numbered envelope is assigned to each patient and is opened by the same research fellow after the first 24 h of treatment.

Patients and their anesthesiologists are blind to treatment assignment. A clinical resident, who is not involved with randomization, is in charge of all clinical evaluations as part of the daily clinical practice.

The envelope is maintained apart from the patient’s case report form and is to be opened in every potentially harmful situation for the patient but only when care providers need to be aware of the patient’s treatment. All ropivacaine and methylprednisolone vials used during the study are registered with their code in order to be tracked and recognized in the future for any issue potentially hindering the validity of data.

At 48 h, morphine PCA is removed, and all patients are treated with Patient-Controlled Intrawound Analgesia (PCA through the wound catheter) with either ropivacaine 0.2 % alone or saline according to the group of randomization up to a maximum of 7 days after surgery. The catheter is removed at the seventh postoperative day or after 36 h of non-use (no self-administered bolus). After PCA removal, rescue additional analgesia is provided by tramadol (100 mg or 50 mg according to patient’s weight of not more than or of more than 50 kg).

All patients are evaluated at the end of surgery, at 3, 6, 12, 24, 36, and 48 h, and daily to the seventh postoperative day or until catheter removal by the Acute Pain Service and twice a day by the surgeons as part of their current clinical practice. Pain values, analgesic consumption, any drug-related side effect, and any catheter-related complication (such as occlusion or dislodgment) are recorded. Patients are also evaluated by surgeons after discharge, as in the current practice: any infection or healing retardation is monitored, together with any pain or sensorial abnormality to be signaled to the pain practitioners. Regardless, the Acute Pain Service investigates pain persistence at 1 and 3 months through a phone call, and patients with PPSP (patients experiencing pain at 1 or 3 months after surgery) are eventually referred to our pain clinic for evaluation and treatment.

### Procedures - genetic study

We will genotype several SNPs in *ADRB2*, *OPRM1*, *COMT*, *UGT2B7*, and *IL-1Ra* genes in all patients by the Taqman SNP Genotyping Assays (Applied Biosystems, Foster City, CA, USA) in the Pain-OMICS core-lab of Azienda Ospedaliera of Parma.

The peripheral blood samples are collected during general anesthesia into EDTA-coated tubes. We extract DNA by using a QIAamp DNA Blood Mini Kit (Qiagen, Hilden, Germany) from 200 μl of blood. DNA obtained are stored at −20 °C for subsequent TaqMan analysis. Amplification is performed in a volume of 5 μl containing 10 ng of genomic DNA, SsoFast Probes Supermix (Bio-Rad Laboratories S.r.l, Segrate, Italy). Cycling and hybridization conditions are set in accordance with the instructions of the manufacturer.

The amplification and genotyping discrimination are performed in a LightCycler 480 Real-Time PCR System (Roche Diagnostics Ltd, Lewes, UK). All assays are used in accordance with the instructions of the manufacturer (Life Technologies, Monza, Italy). The SNP calling is made with LightCycler 480 Endpoint Genotyping software version 1.5.0.39 (Roche Diagnostics Ltd).

### Procedures - inflammatory response study

Inflammatory response will be analyzed on the first 15 patients in each group (for a total of 30 patients). Before the end of surgery, the surgeon inserts a single-use and minimally invasive (diameter of 0.5 mm) micro-dialysis catheter in the fat adjacent to the surgical wound with sterile technique. The catheter has a cutoff membrane (length of 30 mm) of 100,000 Da, which allows the monitoring of inflammation in vivo. The catheter’s characteristics allow extended use without risk of breaking. The catheter will be connected to a slow-rate infusion pump with imposition of 3 μl/min of micro-dialysis liquid, a high molecular weight (30 g dextran 60/1000 ml) substance to facilitate the micro-dialysis that remains inside the catheter. The dialyzed liquid is collected in dedicated tubes directly in the infusion pump. Sampling will be performed at 1, 6, 24, 48, and 72 h. Serum samples at the same time points are collected to compare systemic and local inflammations. All samples are stored at −80 °C until analysis. Concentrations of ILs (IL-1, IL-6, IL-8, and IL-10), interferon-gamma, and tumor necrosis factor-alpha are analyzed in dialyzed wound samples (local inflammation) and in serum (systemic inflammation). Cytokine levels in dialyzed liquid and in serum are tested by enzyme-linked immunosorbent assay (ELISA) by using a commercial kit.

### Procedures - oxidative stress study

Oxidative stress will be analyzed on the first 15 patients in each group (for a total of 30 patients). Patients are sampled before surgery, before morphine bolus, and at 24 and 48 h after surgery.

Urine samples are directly aliquoted and stored at −80 °C; blood samples are collected in EDTA-containing vials and centrifuged within 30 min at 1000×*g* for 15 min at 4 °C to obtain the plasma.

Lipid peroxidation is analyzed through the determination of malondialdehyde with thiobarbituric acid assay in both plasma and urine. The 8-deoxyguanosine and poly(ADP-ribose) polymerase (PARP) are determined by commercial ELISA kits.

### Study outcomes

The primary outcome of the study is to investigate whether a wound infusion of local anesthetic and steroid has a higher analgesic efficacy compared with placebo during 7 days following surgery, assessed as analgesic consumption: the primary endpoint will be the reduction of morphine equivalents required in the first 7 days after surgery.

Secondary outcomes will include the differences in analgesia and side effects within 7 days after surgery. We will investigate any difference in pain at rest and movement (i.e., coughing and deep inspiration) by Numeric Rating Scale (11-point scale from 0 = no pain to 10 = worst imaginable pain), additional analgesic consumption, side effects (postoperative nausea and vomiting, sedation, and any signs of local anesthetic or steroid systemic toxicity), and incidence of persistent post-surgical pain. Differences in terms of wound healing or wound infections will be also assessed up to 3 months to assess any local side effect of steroid infusion. The degree of inflammatory response/oxidative stress between groups will be assessed to analyze the effect of CWI with either treatment or placebo. We plan to analyze the combined effect of some polymorphisms (by using a gene-candidate approach) of genes related to the development of PPSP, pain sensitivity, opioid response, and inflammatory reaction.

### Statistics

Sample size calculation was made on the hypothesis that the average morphine equivalent consumption in patients receiving local anesthetic and steroids CWI will be reduced by 50 % as compared with those in the control group. This assumption comes from (1) our retrospective experience and audits about analgesic consumption in our institution and (2) data from previous literature [27]; they both showed reduction in morphine consumption of 30–50 % in patients receiving local anesthetic wound infusion. Given this assumption, we need 50 patients per group for a power of 80 % and an α error of 5 %; given an attrition rate of 15–20 %, 120 patients are needed in each group.

We will perform an intention-to-treat analysis, and all participants will be analyzed once enrolled. We consider a “dropout” to be any patient not completing the evaluation at 3 months (for any reason).

For descriptive variables, we plan to use mean and standard deviation for normally distributed variables and average interquartile range for skewed distributions.

We will perform group comparison by using the Student’s *t* test or Mann–Whitney test for quantitative variables and Pearson’s chi-squared test for categorical variables. Inflammation, oxidative stress, and genetic background will be assessed to see whether they are biological markers that help modulate post-operative and persistent pain: the difference in inflammatory response and oxidative stress will be analyzed by means in a uni- and multi-variate regression model for repeated measures, including time, group, and their interaction. Variables will be transformed if needed to satisfy model assumptions. Post-hoc comparisons will include within-group changes over time and between-group differences at each assessment. The *P* value will be adjusted for multiple tests. Fisher exact test will be used to evaluate the impact of genetic background. The odds ratio and 95 % confidence interval will be computed with logistic regression. The *P* value to assess statistical significance will be corrected with the Bonferroni correction depending on the number of candidate genes tested.

## Discussion

Historically, care providers have considered pain a perioperative issue able to influence immediate morbidity, but in the last decade greater attention has been placed on the long-term effects of unrelieved postoperative pain, especially PPSP [4, 5]. Postoperative pain is a composed phenomenon, in which different variables have to be taken into account [28]. Therefore, new multidisciplinary studies should address pain in a different perspective, heading toward a pathophysiological approach to the different components of pain perception and not merely to analgesia expressed as a numeric value in the first days after surgery. Unfortunately, not all of these components are known, and therapeutic approaches still need to be improved.

When clinicians deal with acute postoperative pain, the surgical wound is a pivotal target for analgesia; nociceptors located within the surgical incision are the origin of pain stimuli coming from the periphery, and their prolonged activation is one of the causes of central sensitization. Inflammation also is a main determinant of pain; a lot of mediators are increasingly expressed after tissue injury leading to primary hyperalgesia [12], and IL-1 has been linked to pain persistence [14]; thus, a therapeutic approach specifically aiming to reduce the inflammatory response is rational. CWI with non-steroidal anti-inflammatory drugs seems to provide only partial advantages [11] (because they act only on COX-associated mediators, like prostaglandin E), whereas steroids have a wide anti-inflammatory effect and arguably a great efficacy against pain perception and chronicization [8, 16]. In our study, we decided to test the efficacy of CWI with both local anesthetic and steroids in patients undergoing major abdominal surgery, configuring the first phase III clinical trial on continuous deep wound infusion of methylprednisolone.

Peripheral administration should minimize systemic adverse effects by reducing bioavailability and plasma concentration; otherwise, any data about pharmacokinetics and potential side effects of continuous infusion of steroids are available: we therefore decided to exclude patients with any disease potentially precipitating after steroid systemic absorption, primarily diabetes (type 1 or 2).

In regard to local side effects, the main concerns are about wound healing and infections potentially associated with immune-modulatory action of steroids. Most of the existing data deal with high doses of systemic steroids [29, 30]. Up to now, few studies have addressed these issues in patients receiving peripheral steroids: these studies are in the orthopedic setting and investigate only clinical effects of a single-shot administration and provide no evidence of increased infections or difficult healing [29, 30]. We therefore plan to clarify the risk/benefit ratio of continuous administration of methylprednisolone, strictly monitoring wound healing and any potentially harmful effect of CWI.

We decided to treat all the patients with 24-h CWI of ropivacaine and methylprednisolone. This will enlarge the sample size for the phase III trial (120 patients) and will allow us to investigate the differences in locally released cytokines according to suspension of local anti-inflammatory treatment after the first 24 h. This, in our opinion, does not configure a methodological bias, as our primary endpoint is analgesic consumption at 7 days, and we will take care to evaluate any statistically significant difference in morphine consumption at 24 h to eliminate any confounding factor this kind of study design may potentially imply.

Furthermore, an ethical issue arises: because CWI with local anesthetics is already documented as a valuable analgesic strategy [8], switching to a saline infusion after 24 h in the control group may be questioned. Otherwise, placebo control is still a key feature for high-quality clinical trials in the perspective of determining the actual effectiveness of treatments. Furthermore, because we are investigating local inflammation, comparing analgesic efficacy with cytokine analysis will help clarify whether the pain-killing effect of CWI is associated either with methylprednisolone’s action or with a “washout” phenomenon (i.e., on the steroid’s effect on cytokine release rather than on cytokine dilution by fluid infusion).

We decided to analyze both local and systemic inflammatory response for the following reasons. First, systemic inflammation is advocated to be a risk factor for postoperative and persistent pain [5], but few data exist, and failed in the identification of standard values of inflammatory markers as predictors of pain; therefore, we guess our data could configure as a reference for future studies. Second, we want to investigate whether local and systemic cytokine expressions are linked or not, and how. Third, we aim to investigate the effect of a peripheral block on systemic inflammation because previous studies have provided an incomplete understanding [11]. Furthermore, there is evidence of the role of free radicals as a crucial mediator of pain of different etiologies [31–33], and we will also investigate the free-radical formation and correlate these data to the inflammatory response.

The maintenance of CWI up to 7 days could also be questioned because we traditionally consider the first 48 h after surgery to be the most challenging and to be associated with the highest pain values, and most of the studies consider a 2-day infusion after surgery; otherwise, studies on incisional pain have demonstrated that sensitization processes tend to restart after suspension of analgesia [34]. Therefore, an effective nociceptive block should cover the entire perioperative period, aiming to fight pain until it lasts. Use of patient-controlled wound infusion arguably allows prolongation of the analgesic infusion as deemed necessary by the patient, without drug overdosing, and will help quantify the average amount of drugs needed in the first 7 days after major abdominal surgery. This final issue is to be considered carefully because our study is part of a wider one aimed to validate a new nanotech device for pain treatment: this device is being created by bio-engineers and will be tested for bio-compatibility in rats. The final aim is to develop a new analgesic tool to be based on nanotechnology and to be applied on the surgical wound in humans for sustained release of pain medications and growth factors after surgery and to be finally re-absorbed by tissues. In this perspective, our study is the first step of a longer project and is focused on investigating (1) the efficacy and safety of topical methylprednisolone, (2) the average dose of pain medications required in the perioperative period, and (3) the average needed duration of a wound infusion.

With this study, we aim to validate the use of methylprednisolone for CWI to reduce morphine consumption and associated side effects as well as PPSP after abdominal surgery. Moreover, we want to investigate whether genetic polymorphisms might be biomarkers for postoperative pain and opioid consumption. These results should be used to define delivery times and dosages for the new nanotechnology platform we are currently developing for sustained postoperative pain relief to be used in clinical practice and should be able to improve perioperative and long-term outcome for patients.

## Trial status

The trial is currently running, all three centers are currently involved in enrollment, and we have enrolled 46 patients so far.

## Abbreviations

ADRB2: Adrenergic receptor α-2
COMT: Catecholamine-O-methyltransferase
COX: Cyclooxygenase
CWI: Continuous wound infusion
ELISA: Enzyme-linked immunosorbent assay
IL: Interleukin
IL-1Ra: Interleukin-1 receptor antagonist
OPRM1: μ opioid receptor-1
PCA: Patient-controlled analgesia
PPSP: Persistent post-surgical pain
SNP: Single-nucleotide polymorphism
UGT2B7: UDP-glucuronosyltransferase 2B7
